# Supplementary material for: The Systems Biology Research Tool: evolvable open-source software
Source: BMC Syst Biol. 2008 Jun 29;2:55. doi: 10.1186/1752-0509-2-55 (PMC2446383; doi:10.1186/1752-0509-2-55)
Supplement: Additional file 1 — SBRT Archive. An archive of the current version of the Systems Biology Research Tool. [file 1752-0509-2-55-S1.zip › sbrt-1.4.0/doc/developers_guide/api/sbrt/shell/io/MapTextFile.html]

MapTextFile


|  |  |  |
| --- | --- | --- |
| **Overview** \| **Package** \| **Class** \| **Use** \| **Tree** \| **Deprecated** \| **Index** \| **Help** | |  |
| **PREV CLASS**   **NEXT CLASS** | **FRAMES**    **NO FRAMES**     **All Classes** |  |
| SUMMARY: NESTED \| FIELD \| CONSTR \| METHOD | DETAIL: FIELD \| CONSTR \| METHOD |  |


---


## sbrt.shell.io Interface MapTextFile<K,V>

**Type Parameters:**: `K` - the key type.: `V` - the value type.

**All Superinterfaces:**: SbrtFile

**All Known Subinterfaces:**: MapTextInputFile<K,V>, MapTextOutputFile<K,V>

**All Known Implementing Classes:**: AbstractMapInputFile, AbstractMapOutputFile, ComiFile, ComoFile, FluxCapInputFile, ProcessInputFile, RomiFile, RomoFile

---

``` public interface MapTextFile<K,V> extends SbrtFile ```

This interface is used to represent text-based
files that contain maps.

**Author:**
:   This interface was written and documented by
    Jeremiah Wright while in the Wagner lab.

---

| **Method Summary** | |
| --- | --- |
| `MapFormat<K,V>` | `getMapFormat()`             Returns the map format used by this file. |

**Methods inherited from interface sbrt.shell.io.SbrtFile**

`close, getCanonicalName, getOriginalName, isClosed`

**Method Detail**

### getMapFormat

```
MapFormat<K,V> getMapFormat()
```

:   Returns the map format used by this file.

    :   **Returns:**: the map format used by this file.


---


|  |  |  |
| --- | --- | --- |
| **Overview** \| **Package** \| **Class** \| **Use** \| **Tree** \| **Deprecated** \| **Index** \| **Help** | |  |
| **PREV CLASS**   **NEXT CLASS** | **FRAMES**    **NO FRAMES**     **All Classes** |  |
| SUMMARY: NESTED \| FIELD \| CONSTR \| METHOD | DETAIL: FIELD \| CONSTR \| METHOD |  |


---
